# Timing of Pars Plana Vitrectomy in Management of Gunshot Perforating Eye Injury: Observational Study

**DOI:** 10.1155/2016/1487407

**Published:** 2016-10-03

**Authors:** Hammouda Hamdy Ghoraba, Mohamed Amin Heikal, Hosam Osman Mansour, Haithem Mamon Abdelfattah, Emad Mohamed Elgemai, Adel Galal Zaky

**Affiliations:** ^1^Tanta University, Tanta, Egypt; ^2^Magrabi Eye Hospital, Tanta, Egypt; ^3^Benha University, Benha, Egypt; ^4^Al-Azhar University, Domyat, Egypt; ^5^Benha Teaching Hospital, Benha, Egypt; ^6^Damanhour Teaching Hospital, Damanhur, Egypt; ^7^Menoufia University, Shebin El Kom, Egypt

## Abstract

The aim of this study is to report the difference in either anatomical or functional outcome of vitreoretinal intervention in cases of gunshot perforating eye injury if done 2–4 weeks or after the 4th week after the original trauma. Patients were treated with pars plana vitrectomy and silicon oil. Surgeries were performed in the period from February 2011 until the end of December 2014. 253 eyes of 237 patients were reviewed. 46 eyes were excluded. 207 eyes of 197 patients were analyzed. The included eyes were classified based on the timing of vitrectomy in relation to the initial trauma into two groups: 149 eyes (the first group) operated on between the 3rd and the 4th week and 58 eyes (the second group) operated on after the 4th week after the trauma. Following one surgical intervention, in the first group, attached retina was achieved in 93.28% of patients. In the second group, attached retina was achieved in 96.55% of patients. All RD cases could be attached by a second surgery. Visual acuity improved in 81.21% of patients, did not change in 15.43% of patients, and declined in 3.35% of patients. In the second group, visual acuity improved in 81.03% of patients, did not change in 12.06% of patients, and worsened in 6.89% of patients. There was no statistically significant difference between the two groups in either anatomical or functional results. We recommend interfering before the 5th week after the trauma as retinal detachment is encountered more in cases operated on after the 4th week. The visual outcome depends on the site of entry and exit (the route of gunshot).

## 1. Introduction

Mechanical injuries of the globe (open and closed) are classified according to Pieramic et al. [1] system that relies on four variables: type of injury, grade of injury, pupillary response, and zone of injury [1]. Open globe injuries are a common and often preventable cause of permanent visual impairment and visual loss [2]. Perforating ocular injuries are “through-and-through” globe defects with entry and exit sites. This is in contrast to penetrating injuries, which have a point of entry into the globe but no exit wound [3, 4].

Histopathological studies revealed that posterior vitreous detachment (PVD) usually occurred at 1 to 2 weeks after trauma [5]. The peripheral tractional retinal detachment then developed between 7 and 11 weeks, due to contractile fibrovascular ingrowth from the wound along the vitreous scaffold to the vitreous base and from preretinal membranes in the peripheral and equatorial retina. The end result at 4 months was tractional total retinal detachment and fibrous cyclitic, epiretinal, and subretinal membranes [6]. If not operated on it ends by phthisis bulbi [7].

Experimental and clinical studies suggest that, among all types of globe wounds, perforating injuries have the worst prognosis [8–10]. The factors limiting visual recovery include direct injury to the optic nerve or macula, intraocular scarring and fibrosis with secondary retinal detachment, and severe ocular disorganization [11, 12]. Several predictive factors affect the prognosis for final visual acuity [13].

Previous reports showed that vitreoretinal surgery in perforating injury prevents phthisis bulbi and achieves some functional result [7]. There is controversy to do vitreoretinal surgery early days or weeks after repair of the primary injury [14] or to use encircling scleral band or no [15].

The aim of this study was to compare the results of PPV during the 3rd to 4th or later than the 4th week after trauma in gunshot perforating eye injury.

## 2. Patients and Methods

This is a retrospective observational study of 207 eyes of 197 patients with perforating eye injuries caused by gunshots treated by pars plana vitrectomy and silicon oil with or without buckle.

Surgeries were performed in the period from February 2011 to the end of December 2014 during the period of political instability in Egypt. All surgeries were performed by a single surgeon (HG) at a single center.

All patients had preoperative evaluation included: best corrected visual acuity, intraocular pressure measurement, anterior segment examination using the slit lamp, and dilated fundus examination using indirect ophthalmoscope if the media were clear. The ocular trauma score (OTS) was retrospectively calculated.

Investigations done were B/scan ultrasonography and Computerized Tomography (CT) to locate the gunshot and for medicolegal aspect. Visual Evoked Potential (VEP) was requested for cases with no light perception to justify no surgical intervention.

*Inclusion Criteria*. Perforating gunshot ocular injury with at least light perception vision with minimum follow-up 6 months after the last surgical intervention was the inclusion criterion.

*Exclusion Criteria*

 Patients with visual acuity of no light perception
 Patients with retained intraocular foreign body (gunshot)
 Patients with endophthalmitis
 Patients with follow-up less than 6 months after the last surgical intervention.

### 2.1. Surgical Procedures

Primary repair was done elsewhere in all cases in the same day of trauma. Our plan for vitreoretinal intervention in such cases was to operate on at least 2 weeks after the primary repair to allow entry wound healing, suprachoroidal hemorrhage to liquefy if present and posterior vitreous detachment to occur. In this series, some factors made the time of intervention variable; for example, patients with scleral and limbal entry were operated on during the 3rd week after trauma. Patients with a central corneal wound or suprachoroidal hemorrhage were operated on in the 4th week from injury to allow more time for proper wound healing. Patients referred after the 4th week of trauma were operated on once they were presented to us.

The surgical technique was the same in all cases and was done by the same surgeon (HG). Three-port pars plana vitrectomy (PPV) was done using conventional 20 gauges or transconjunctival cannulated 20 or 23 gauges with or without scleral buckling.

Lensectomy using fragmentation or vitrectomy probe was done if there was cataract interfering with proper visualization or if lens touch occurred; otherwise the lens was spared.

A central vitrectomy was performed until central PVD was achieved if not already present. Perfluorocarbon (PFC) was injected to flatten the retina in cases with retinal detachment, to get a better view, to elevate the residual vitreous and to displace subretinal hemorrhage anteriorly.

Vitrectomy was completed as safe as possible anteriorly leaving an amalgam of tissue around the exit site to prevent PFC, air, and silicon from escaping into the orbit. No chorioretinectomy was done in those cases.

Vitrectomy under air was used frequently in the presence of bleeding. Laser was applied to any retinal break, 360 degrees and around the exit site if it was outside the macula. Air PFC exchange was done followed by silicon oil injection of 2,000 or 5,000 cSt. The bright illumination and wide field visualization systems allowed us to do all surgeries without the need of penetrating keratoplasty.

All patients were examined in the 1st postoperative day. Fundus examination and color fundus photography were done if possible. Postoperative follow-up was scheduled at 1 week, 3 weeks, 6 weeks, and then every 8 weeks.

In cases of development of retinal detachment after the primary surgery, the surgical procedure consisted of silicon oil removal, triamcinolone-assisted removal of any residual vitreous cortex, and removal of epiretinal membrane if involving the macula. Relaxing retinotomy was done in cases with excessive retinal proliferation preventing retinal attachment or subretinal S.O. PFC was injected. Laser was added to any break and to the edge of retinotomy followed by air PFC exchange. Silicon oil 5,000 cSt was injected at the end of surgery.

Statistical tests used are mean, standard deviation, chi-square test, and *P* value. *P* value was considered significant if <0.05. Statistical analysis was performed using a commercially available statistical software package (SPSS for windows, version 20).

## 3. Results (Tables 1 [Table tab2] [Table tab3] [Table tab4] [Table tab5]–6, Figures 1 [Fig fig2] [Fig fig3] [Fig fig4] [Fig fig5]–6)

253 eyes of 237 patients were reviewed. Excluded cases were 9 eyes due to no light perception at their presentation, 24 eyes with intraocular gunshot, and 13 eyes due to short follow-up (less than 6 months). 207 eyes of 197 patients were analyzed.

The included eyes were classified according to the duration between trauma and vitreoretinal intervention into two groups; the first group included 149 eyes in which vitreoretinal surgery was done between the 3rd and the 4th week after injury and the second group included 58 eyes in which vitreoretinal surgery was done after the 4th week after the injury.

In the first group, 129 patients (88.35%) were males and 17 patients (11.65%) were females. The age ranged from 4 to 48 years with mean ± standard deviation (25.58 ± 8.2 years). In the second group, 41 patients (80.39%) were males and 10 patients (19.61%) were females. The age ranged from 4 to 55 years with mean ± standard deviation (26.57 ± 11.23 years).

Preoperative visual acuity was distributed among the two groups as follows: in the first group, visual acuity was LP in 47 eyes (31.54%), HM in 85 eyes (57.04%), CF at 1 m in 10 eyes (6.71), 20/200 in 4 eyes (2.68%), and 20/100 in 3 eyes (2.01%). In the second group, visual acuity was LP in 29 eyes (50%), HM in 25 eyes (43.10%), CF at 1 m in 2 eyes (3.44%), 20/200 in 1 eye (1.72%), and 20/100 in 1 eye (1.72%).

In the first group, 38.92% of entry sites were corneal, 40.94% were scleral, and 20.13% were limbal. In the second group, 44.82% of entry sites were corneal, 32.75% were scleral, and 22.41% were limbal.

Mean preoperative ocular trauma score (OTS) was 43.96 ± 12.64 in group 1 and 41.72 ± 12.94 in group 2. No statistically significant difference was found between both groups (*P* = 0.622).

The exit site was found at the macula in 49 eyes (32.88%) in the first group and 10 eyes (17.24%) in the second group. Optic nerve exit was observed in 21 eyes (14.09%) in the first group and 9 eyes (15.51%) in the second group. The exit site other than macula and optic nerve was present in 79 eyes (53.02%) in the first group and 39 eyes (67.24%) in the second group.

Retinal detachment and retinal incarceration were seen more frequent in group two with statistically significant difference (*P* values 0.001 and 0.032, resp.).

Most of the cases with retinal detachment in the two groups were accompanied with vitreous hemorrhage (78.26% in group 1 and 53.57% in group 2).

Operative findings are mentioned in Table 2.

By one operation anatomical results in the first group revealed attached retina in 139 eyes (93.28%) and 10 eyes (6.72%) developed RD. In the second group attached retina was achieved in 56 eyes (96.55%) and 2 eyes (3.45%) developed RD. All retinal detachment cases could be reattached by a second surgery. No eyes developed phthisis bulbi during the follow-up period.

*There was no statistically significant difference between the two groups regarding anatomical results*.

We did not notice any escape of either S.O. or PFCL into the orbit either during or after surgery. The reported postoperative complications were presented in Table 3.

In the first group,* postoperative VA* was LP in 12 eyes (8.05%), HM in 52 eyes (34.89%), CF at 1 m in 38 eyes (25.5%), 20/200 in 38 eyes (25.5%), and 20/100 in 9 eyes (6.04%). Visual acuity improved in 121 eyes (81.21%), unchanged in 23 eyes (15.43%), and declined in 5 eyes (3.35%).

In the second group, VA was LP in 4 eyes (6.89%), HM in 26 eyes (44.82%), CF at 1 m in 13 eyes (22.41%), 20/200 in 12 eyes (20.68%), and 20/100 in 3 eyes (5.17%). Visual acuity improved in 47 eyes (81.03%), unchanged in 7 eyes (12.06%), and worsened in 4 eyes (6.89%).

*There was no statistically significant difference between the two groups regarding functional results*.

The postoperative anatomical and functional results were shown in Table 4.

The best corrected visual acuity improved in the two groups as compared to the preoperative VA (Table 5).

The main cause of low visual outcome was the central route of the gunshot, central corneal entry, and macular or optic nerve exit. This was shown in Tables 6(a), 6(b), 6(c), and 6(d).* There was no statistically significant difference between the two groups*.

## 4. Discussion

Perforating injuries of the globe account for a small portion of open globe injuries [2]. The incidence increased in Egypt since January 2011 due to political instability. The standard approach to treating perforating injuries is primary repair to restore the structural integrity of the globe at the earliest opportunity [2]. Previous reports showed the benefit of vitreoretinal surgery in such cases in preventing phthisis bulbi and achieving some visual result [7].

Controversy remains about the best timing of secondary intervention [14]. There are 3 opinions regarding the timing of PPV in such cases: early vitrectomy within 1 to 3 days [16] and delayed vitrectomy between 7 and 14 days [3, 17] and more than 14 days [18, 19].

The argument for early vitrectomy within 1 to 3 days is to remove all proinflammatory factors before the beginning of fibrosis. The counterargument is that operating on an acutely traumatized eye can have unpredictable findings with a higher likelihood of continued hemorrhage. Suprachoroidal hemorrhage usually is not liquefied, making drainage difficult. Vitrectomy is also more challenging because a spontaneous PVD usually does not develop during this period, especially in young patients [20].

Waiting for 7–14 days after the primary repair allows spontaneous PVD to occur and a more thorough examination with ultrasonography to determine whether the eye is salvageable based on the intraocular anatomic status [20].

In our report, 253 eyes of 237 patients were reviewed. We excluded 9 eyes with no light perception at their presentation, 24 eyes with an intraocular foreign body, and 13 eyes due to short follow-up (less than 6 months from the last surgical intervention). 207 eyes of 197 patients are analyzed.

Patients with scleral and limbal entry were operated on during the 3rd week after trauma. Patients with a central corneal wound or suprachoroidal hemorrhage were operated on in the 4th week from injury to allow more time for proper wound healing. Patients referred after the 4th week of trauma were operated on once they were presented to us. Mean preoperative ocular trauma score (OTS) was 43.96 ± 12.64 in group 1 and 41.72 ± 12.94 in group 2. No statistically significant difference was reported between both groups (*P* = 0.622).

However the OTS in perforating trauma might not be accurate due to the presence of hyphema, dense vitreous hemorrhage, and/or RD which affected the evaluation of afferent pupillary defect (APD) necessary for scoring of the trauma [21]. Also there might be a bias in case selection by the referring physician. It could be that patients with severe injury were never referred from the ophthalmologist. The higher frequency of retinal detachment (RD) and retinal incarceration and lower frequency of macular exit sites in the second cohort are consistent with the possibility that the cohorts are not balanced.

Anatomical results in the first group revealed attached retina in 139 eyes (93.28%) and 10 eyes (6.72%) developed RD. In the second group attached retina was achieved in 56 eyes (96.55%) and 2 eyes (3.45%) developed RD. All retinal detachment cases could be reattached by a second surgery. No eyes developed phthisis bulbi during the follow-up period.

During primary PPV we found RD more in cases operated after the 4th week. It was related to the exit site. Most of cases with retinal detachment in both groups were accompanied with vitreous hemorrhage. This agrees with previous reports [5].

*There was no statistically significant difference between the two groups regarding the anatomical results*.

Pieramic et al. [1] recommended early vitrectomy within 72 hours after injury. While Vatne and Syrdalen [18] could not identify a beneficial effect of early vitrectomy after injury. They reported that the cases operated on more than 2 weeks gained anatomical success (18 eyes of 27 eyes) better than cases operated on earlier than 2 weeks (7 eyes of 14 eyes).

In their report, Abrams et al. [8] recommended vitrectomy 2 weeks after primary repair but no details about the definite time in their series. Abd EL Alim [22] reported different times of vitreoretinal intervention after the primary repair (less than 1 week in 5%, 2-3 weeks in 73%, and 3–6 weeks in 22% of patients). He excluded cases with corneal entry and macular and optic nerve exit and did not compare the anatomical and functional results between them.

The advances in visualization (wide field system, brighter light) and better cutting technique may be the causes of better results in our series as compared with previous results.

In this series, the best corrected visual acuity improved in the two groups as compared preoperatively. Analysis of the two groups shows the following.

In the first group visual acuity improved in 121 eyes (81.21%), unchanged in 23 eyes (15.43%), and declined in 5 eyes (3.35%).

In the second group visual acuity improved in 47 eyes (81.03%), unchanged in 7 eyes (12.06%), and worsened in 4 eyes (6.89%).

*There was no statistically significant difference between the two groups regarding the visual acuity*.

The final visual acuity in our study depends mainly on the location of the entry site and the exit site (macular or optic nerve). The cases with a central corneal wound and macular and optic nerve exit had bad visual prognosis in our series.

Hermsen [19] reported that final visual acuity in his series is similar in patients who had early vitrectomy (1–14 days) and those who underwent vitrectomy after 14 days. The best results were achieved when vitrectomy was performed between 15 and 30 days following injury.

Vatne and Syrdalen [18] reported that final result depends mainly on the severity of the primary injury.

Ramsay et al. [10] reported that the surgical success was related to initial visual acuity and the extent of vitreous hemorrhage. These factors in our opinion reflect the severity of trauma.

The drawbacks of our report are the retrospective nature and the patients with late intervention after the 4th week after trauma were analyzed as one group (group 2) without dividing it into different times of referral, for example (4–6 weeks, 6–8 weeks, 8–10 weeks, etc.).

Although statistical analysis showed no difference between the two groups, we think that pars plana vitrectomy is better to be done 2 weeks after injury to allow the entry site to heal and PVD to develop and suprachoroidal hemorrhage to liquefy. It is better not to be done more than 4 weeks after the primary injury to avoid fibrous ingrowth from the entry site to the exit site with possible retinal detachment and incarceration into the entry or exit site. Intervention should be taken case by case.

## 5. Conclusion

In gunshot perforating eye injury, there was no statistical difference between cases operated on during the 3rd or the 4th versus after the 4th week after primary repair in either the anatomical or functional results. However, we recommend interfering before the 5th week after the trauma as retinal detachment is encountered more in cases operated on after the 4th week. The visual outcome depends on the site of entry and exit (the route of gunshot).

## Figures and Tables

**Figure 1:**
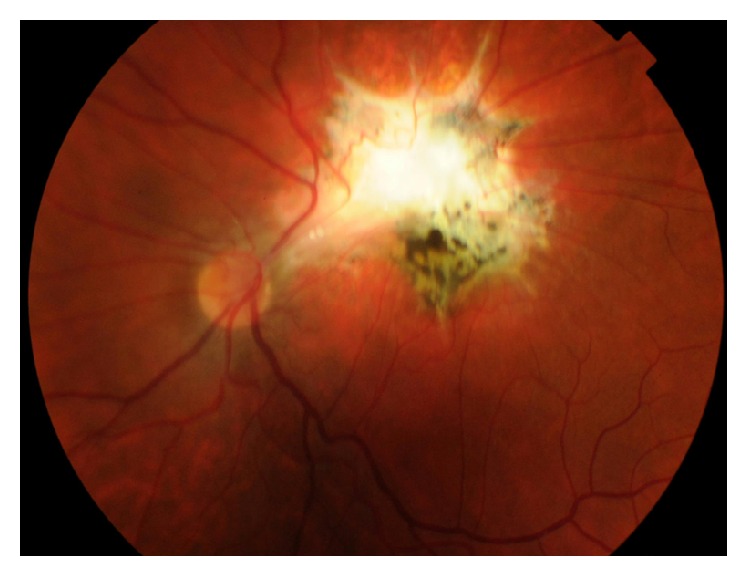
Color photo of left eye showing parafoveal exit with macular dragging.

**Figure 2 fig2:**
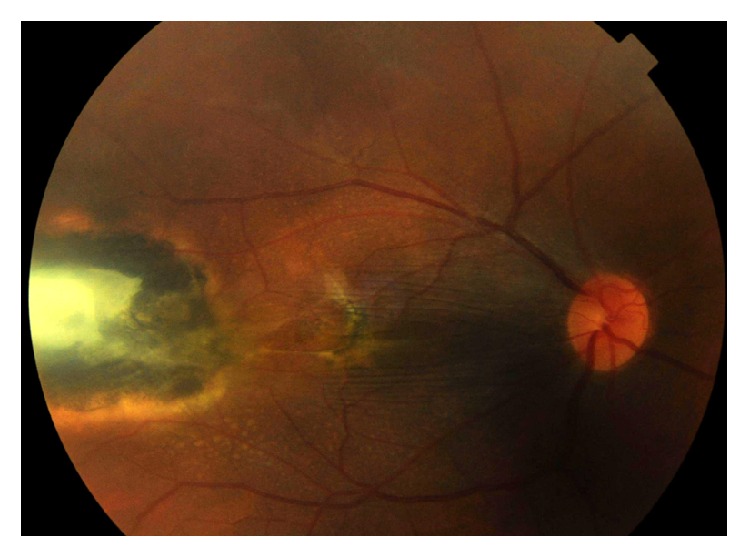
Color photo of the right eye showing temporal exit with macular dragging.

**Figure 3 fig3:**
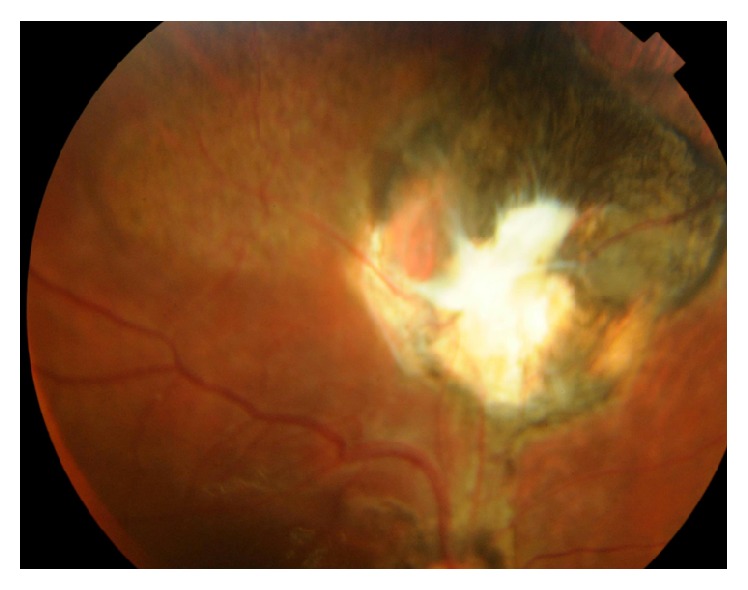
Color photo of the right eye showing superior exit.

**Figure 4 fig4:**
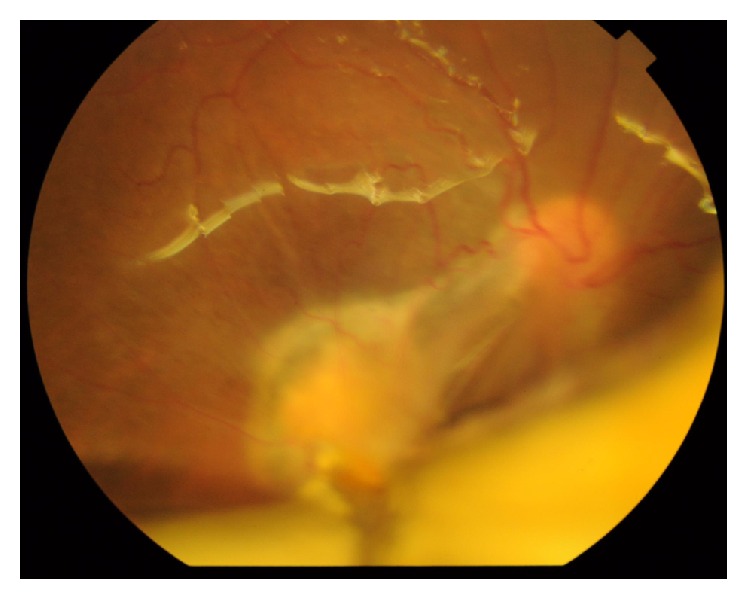
Color photo of right eye showing retinal incarceration at the exit site and lower RD under silicon oil.

**Figure 5 fig5:**
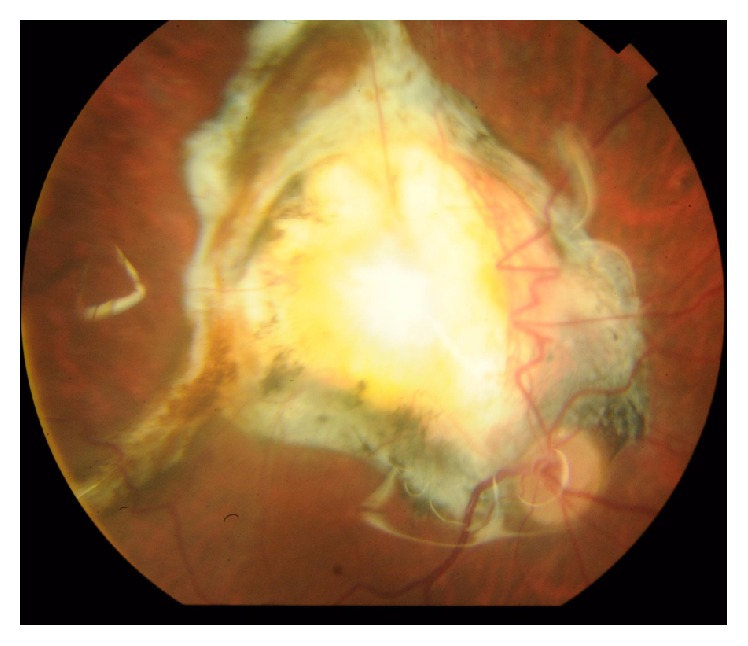
Color photo of right eye showing macular exit.

**Figure 6:**
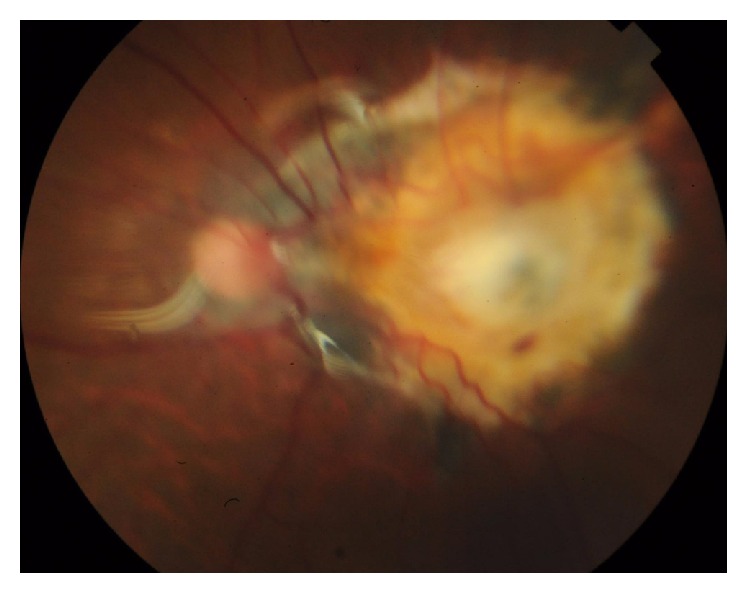
Color photo of left eye showing macular exit with retinal incarceration.

**Table 1:** Preoperative data of the patients.

		Group 1 2 w–4 w (149)		Group 2 >4 w (58)		*X* ^2^	*P* value
		*N*	%	*N*	%		
BCVA^*∗*^ preoperatively	LP^*∗∗*^	47	31.54	29	50	6.34	0.175
	HM^†^	85	57.04	25	43.10		
	CF1m^‡^	10	6.71	2	3.44		
	20/100–20/200	4	2.68	1	1.72		
	20/40–20/100	3	2.01	1	1.72		

Anterior segment findings	Hyphema	21	14.09	3	5.17	4.746	0.577
	Lens subluxation	6	4.02	2	3.44		
	Aphakia	9	6.04	2	3.44		
	Cataract	25	16.77	10	17.24		
	Vitreous in anterior chamber	16	10.73	7	12.06		
	Anterior synechia	4	2.68	1	1.72		

Entry site	Corneal	58	38.92	26	44.82	1.186	0.553
	Scleral	61	40.94	19	32.75		
	Limbal	30	20.13	13	22.41		

^*∗*^BCVA: best corrected visual acuity.

^*∗∗*^LP: light perception.

^†^HM: hand motion.

^‡^CF: counting finger.

*P* is significant if <0.05.

**Table 2 tab2:** Intraoperative findings and exit site of the studied groups.

		Group 1 2–4 weeks (149)		Group 2 >4 weeks (58)		*X* ^2^	*P* value
		*N*	%	*N*	%		
Intraoperative findings^*∗*^	Suprachoroidal hemorrhage	12	8.05	2	3.44	1.404	0.236
	Dense vitreous hemorrhage	45	30.20	15	25.86	0.382	0.537
	Retinal detachment	23	15.43	25	43.10	17.942	0.001
	Retinal incarceration	6	4.02	7	12.06	4.588	0.032
	Retinal fold	3	2.01	4	6.89	3.047	0.081
	Subretinal fibrosis	1	0.67	2	3.44	2.254	0.133
	Submacular hemorrhage	7	4.69	2	3.44	0.157	0.692
	Choroidal detachment	6	4.02	1	1.72	0.678	0.410
	Choroidal rupture	3	2.01	1	1.72	0.018	0.892

Exit site	Macular	49	32.88	10	17.24	5.125	0.077
	Disc	21	14.09	9	15.51		
	Others	79	53.02	39	67.24		

^*∗*^For intraoperative findings comparison was made for each item separately as some cases have many findings.

**Table 3 tab3:** Postoperative complications.

	Group 1		Group 2		*X* ^2^	*P* value
	*n* = 149	%	*n* = 58	%		
Cataract	19	12.75	10	17.24	7.115	0.715
Hypotony	5	3.35	4	6.89		
Recurrent retinal detachment	7	4.69	2	3.44		
Retinal proliferation	10	6.71	6	10.34		
Macular pucker	18	12.08	5	8.62		
Corneal scar	21	14.09	6	10.34		
Band keratopathy	5	3.35	3	5.17		
Persistent high IOP^*∗*^ despite treatment	5	3.35	2	3.44		
Silicon oil in A/C^*∗∗*^	2	1.34	2	3.44		
Subretinal fibrosis	8	5.37	5	8.62		

^*∗*^IOP: intraocular pressure.

^*∗∗*^A/C: anterior chamber.

*P* is significant if <0.05.

**Table 4 tab4:** Postoperative anatomical and functional results in both groups.

		Group 1 2–4 w (149)		Group 2 >4 w (58)		*X* ^2^	*P* value
		*N*	%	*N*	%		
Anatomical success	Attached retina by one operation	139	93.28	56	96.55	0.814	0.367
	Attached retina by 2nd operation	149	100	58	100		

Postoperative BCVA^*∗*^	LP^*∗∗*^	12	8.05	4	6.89	1.78	0.77
	HM^†^	52	34.89	26	44.82		
	CF1m^‡^	38	25.5	13	22.41		
	20/200	38	25.5	12	20.68		
	20/100	9	6.04	3	5.17		

^*∗*^BCVA: best corrected visual acuity.

^*∗∗*^LP: light perception.

^†^HM: hand motion.

^‡^CF: counting finger.

*P* is significant if <0.05.

**Table 5 tab5:** Comparison between pre- and postoperative BCVA in both groups.

BCVA^*∗*^	Preoperative 207 eyes		Postoperative 207 eyes		*X* ^2^	*P* value
	Number	%	Number	%		
LP^*∗∗*^	76	36.71	16	7.73	109.5	0.001
HM^†^	110	53.14	78	37.68		
CF 1 m^‡^	12	5.79	51	24.63		
20/100–20/200	5	2.41	50	24.15		
20/40–20/100	4	1.93	12	5.79		

^*∗*^BCVA: best corrected visual acuity.

^*∗∗*^LP: light perception.

^†^HM: hand motion.

^‡^CF: counting finger.

*P* is significant if <0.05.

**(a):** 

BCVA^*∗*^ postoperatively	Corneal entry		Macular exit		Disc exit		*X* ^2^	*P* value
	Number	%	Number	%	Number	%		
LP^*∗∗*^	9	10.71	8	13.55	11	36.66	17.295	0.002
HM^†^	47	55.95	34	57.62	18	60		
CF 1 m^‡^	28	33.33	17	28.81	1	3.33		

^*∗*^BCVA: best corrected visual acuity.

^*∗∗*^LP: light perception.

^†^HM: hand motion.

^‡^CF: counting finger.

*P* is significant if <0.05.

**(b):** 

BCVA^*∗*^ postoperatively	Group 1 = 58		Group 2 = 26		*X* ^2^	*P* value
	Number	%	Number	%		
LP^*∗∗*^=	8	13.79	1	3.84	2.597	0.273
HM^†^	33	56.89	14	53.84		
CF 1 m^‡^	17	29.31	11	42.30		

^*∗*^BCVA: best corrected visual acuity.

^*∗∗*^LP: light perception.

^†^HM: hand motion.

^‡^CF: counting finger.

*P* is significant if <0.05.

**(c):** 

BCVA^*∗*^ postoperatively	Group 1 = 49		Group 2 = 10		*X* ^2^	*P* value
	Number	%	Number	%		
LP^*∗∗*^=	7	14.3	1	10	0.757	0.685
HM^†^	27	55.1	7	70		
CF 1 m^‡^	15	30.6	2	20		

^*∗*^BCVA: best corrected visual acuity.

^*∗∗*^LP: light perception.

^†^HM: hand motion.

^‡^CF: counting finger.

*P* is significant if <0.05.

**(d):** 

BCVA^*∗*^ postoperatively	Group 1 = 21		Group 2 = 9		*X* ^2^	*P* value
	Number	%	Number	%		
LP^*∗∗*^	10	47.6	1	11.1	4.507	0.105
HM^†^	10	47.6	8	88.9		
CF 1 m^‡^	1	4.8	0	0		

^*∗*^BCVA: best corrected visual acuity.

^*∗∗*^LP: light perception.

^†^HM: hand motion.

^‡^CF: counting finger.

*P* is significant if <0.05.
